# Academic Stress, Academic Self-efficacy, and Psychological Distress: A Moderated Mediation of Within-person Effects

**DOI:** 10.1007/s10964-023-01770-1

**Published:** 2023-03-30

**Authors:** Sara Madeleine Kristensen, Torill Marie Bogsnes Larsen, Helga Bjørnøy Urke, Anne Grete Danielsen

**Affiliations:** 1 Department of Health Promotion and Development, University of Bergen, Bergen, Norway; 2 Department of Education, University of Bergen, Bergen, Norway

**Keywords:** Academic stress, Academic self-efficacy, Psychological distress, Gender differences, Random intercept cross-lagged panel model

## Abstract

Previous research has largely failed to separate the between- and within-person effects in the longitudinal associations between academic stress, academic self-efficacy, and psychological distress (symptoms of anxiety and depression). Filling this research gap, this study investigated if academic self-efficacy mediated the relationship between academic stress and psychological distress at the intraindividual level during 3 years of upper secondary school. Gender moderation was also examined in the hypothesised model. The present sample consisted of 1508 Norwegian adolescents (baseline M age = 16.42; 52.9% high perceived family wealth; 70.6% Norwegian-born). The random intercept cross-lagged panel model results indicated (1) positive and time-invariant direct effects from academic stress to psychological distress, (2) academic self-efficacy partially mediated these effects, and (3) psychological distress impacted later academic stress. Academic stress was more strongly related to academic self-efficacy and psychological distress at the interpersonal level for boys, while the intraindividual impact of academic stress on psychological distress was stronger for girls. The study findings might have implications for school-based implementation strategies and theoretical development.

## Introduction

School-related stress affects young people’s quality of life (Berdida & Grande, 2022). Studies show that academic stress (Högberg et al., 2020), including demands and pressure from school (Wiklund et al., 2012) and school-related worry (Sweeting et al., 2010), impacts psychological distress (i.e. symptoms of anxiety and depression: Drapeau et al., 2012; Mirowsky & Ross, 2002) (Torsheim & Wold, 2001) over and beyond previous depressive symptoms (Murberg & Bru, 2005) on an interpersonal, between-person level. However, the intraindividual (i.e. within-person) relationship between academic stress and psychological distress, including relevant explanatory mechanisms and moderators, has largely been ignored. This study employs a moderated random intercept cross-lagged panel model (RI-CLPM) to examine the intraindividual, longitudinal associations between academic stress, academic self-efficacy, and psychological distress in a cohort of upper secondary school students.

During late secondary school, people experience increasing academic pressure from significant adults such as parents (Deb et al., 2015) and teachers (Song et al., 2015). In addition, comparing oneself to and competing with peers intensifies during this period (Eccles et al., 2003), and a series of final examinations that decide future work and educational prospects are on the horizon. In other words, students experience many day-to-day hassles related to their education, such as different pressures and demands to perform well academically during late secondary school (Dewald et al., 2014; Pascoe et al., 2020), and stressful feelings (Leonard et al., 2015; McGraw et al., 2008; Moeller et al., 2020). How adolescents experience stress is highly individual and varies in terms of duration and intensity (Moksnes, Byrne, et al., 2010). Motivation, performance, and well-being can increase if stressors feel challenging due to goal relevance and manageability, resulting in positive stress (eustress: Selye, 1974) (Travis et al., 2020). However, if people lack resources to cope with the various pressures and demands, the stressors are perceived as threatening and can be detrimental to psychological health and well-being (Murberg & Bru, 2005). When adolescents cannot handle a situation, negative stress and accompanying adverse feelings arise (Lazarus, 1966; Sarafino & Smith, 2022).

An increasing secular trend of adolescent psychological distress has been observed during the past decades, internationally (Collishaw, 2015) and particularly in northern Europe (Potrebny et al., 2017) and Norway (von Soest & Wichstrøm, 2014). In Norway, adolescent psychological distress has approximately doubled from 2006 to 2019, increasing from 15 to 30% (Krokstad et al., 2022). A recent study found that academic stress partly explains the rising trend of psychological distress during adolescence (Högberg et al., 2020). The ‘educational stressors hypothesis’ has been put forth as a possible explanation for this association (West & Sweeting, 2003). The educational stressors hypothesis argues that there is a societal development of increasing emphasis on and value of educational attainment, which comes with an increase in school-related stressors (West & Sweeting, 2003). The rising pressure to perform academically and a more prominent focus on normative testing are accompanied by adverse experiences associated with being evaluated, negatively affecting young people’s health (Karvonen et al., 2005). Girls are more likely to experience stress due to these pressures and demands because they place more value on schoolwork and are more susceptible to stressors in their educational environment than boys (Landstedt et al., 2009; Schraml et al., 2011).

Academic self-efficacy (i.e. a person’s belief regarding their capabilities to perform academically: Bandura, 1997) might constitute an explanatory mechanism in the relationship between academic stress and psychological distress (Lazarus, 2006). When people perceive their school-and homework as stressful, their academic self-efficacy might decrease due to the adverse affective state that characterises the negative evaluation (Bandura, 1997). In support of this assumption, studies indicate that school-related stress negatively impacts academic self-efficacy (McKay et al., 2014; Ye et al., 2018). Further, low academic self-efficacy has been established as a predictor of psychological distress cross-sectionally (Karademas & Kalantzi-Azizi, 2004) and longitudinally (Bandura et al., 1999). A reduction in academic self-efficacy might impede individuals’ ability and drive to handle the academic pressures, demands, and difficulties that instigated stressful feelings in the first place, which could result in negative emotions. If individuals do not believe in their academic capabilities enough to cope with their perceived academic stress, feelings of hopelessness and anxiety are promoted (Flett et al., 2011).

### Self-efficacy and the Transactional Theory of Stress and Coping

Lazarus and Folkman (1984) argue that people continuously go through primary and secondary cognitive appraisals, evaluating their situations and the resources available to handle them. A primary appraisal concerns the personal implications of a situation. In late secondary school, students continuously appraise their workload, namely if their school- and homework have implications for their personal well-being. There are three types of situational implications: irrelevant, benign-positive, and stressful (Lazarus & Folkman, 1984, p. 32). A stressful appraisal concerns feelings of harm/loss, threat, or challenge. Feelings of threat and challenge are most relevant to evaluating school- and homework as an implication for personal well-being. *Threat* concerns anticipation of loss or harm, such as being unable to do school- and homework and consequently receiving poor grades. A *challenge* is a positive situation that could lead to personal growth, such as favourable consequences for school success. Students who evaluate their school-and homework as challenging likely experience eagerness, excitement, and exhilaration. On the other hand, students who consider their school workload threatening focus on the potential harms of the situation and characteristically experience negative emotions (Lazarus & Folkman, 1984).

When students perceive their school- and homework as stressful, they must do something to cope (Lazarus & Folkman, 1984). In this case, the second appraisal becomes salient and intricately interacts with the primary appraisal to shape individuals’ emotional reactions (Lazarus & Folkman, 1984). The second appraisal is an evaluation of whether the individual can manage the stressful situation. In other words, what biological, social, and cognitive resources are available to meet and cope with the contextual demands? An example of this evaluation is context-specific self-efficacy (Lazarus, 2006). Perceiving a situation as a threat might negatively inform self-efficacy through the affective/physiological state experienced in the specific setting (Bandura, 1997). The stressful reaction to school- and homework might decrease self-efficacy in the same context (i.e. academic self-efficacy), resulting in increased psychological distress (Bandura, 1997). In contrast, if the academic workload is perceived as challenging, academic self-efficacy might increase due to the positive feelings associated with school-and homework, thus reducing psychological distress.

The transactional relationship between stress, coping, and emotions is a complex system, assumed to be recursive (Lazarus & Folkman, 1987). In other words, a precursor might become an outcome and vice versa as time progresses. Therefore, in addition to the assumed associations described above, it could be beneficial to investigate the possible recursive effects over time. Specifically, psychological distress might simultaneously be an outcome and an antecedent of academic stress and academic self-efficacy. Similarly, academic self-efficacy may be an outcome and precursor of academic stress. For example, psychological distress increases stress in general (Bandura, 1997; Hammen, 2005, 2020) and reduces academic self-efficacy (Bandura, 1997; Grøtan et al., 2019; Usher & Pajares, 2008), which can result in heightened academic stress (Chee et al., 2019; Chemers et al., 2001).

#### Moderating effects

According to Lazarus and Folkman (1984, p. 22), people in different groups have varying degrees of vulnerability and sensitivity to stressors and their understanding and response to them. In support of this assumption in an academic setting, Ye et al. (2018) found that gender moderated the association between academic stress and later academic self-efficacy. Specifically, they found that the association between academic stress and later academic self-efficacy was more salient for girls than boys. Moreover, studies imply that the relationship between academic stress and psychological distress is stronger for girls than boys in secondary school students (Liu & Lu, 2012; Moksnes, Moljord, et al., 2010). There is a lack of studies on the possible gender moderation of the relationship between academic self-efficacy and psychological distress. However, many studies have found gender differences in academic self-efficacy, wherein boys generally report higher levels than girls (for an overview, see Huang, 2013). These findings might imply the existence of gender differences in the association between academic self-efficacy and other factors.

## Current Study

There is a lack of research on the longitudinal relationship between academic stress and psychological distress within adolescents. This study investigates the association between academic stress, academic self-efficacy, and psychological distress on an inter- and intrapersonal level throughout upper secondary school. Additionally, gender differences in these relationships are investigated. The following hypotheses are based on previous research and the assumptions of the transactional theory of stress and coping. First, academic self-efficacy will be negatively related to academic stress and psychological distress, and academic stress and psychological distress will be positively related on an interpersonal level (hypothesis 1). Second, fluctuations in academic stress will predict similar fluctuations in concurrent psychological distress (hypothesis 2). Third, fluctuations in academic self-efficacy will partially explain the association between the fluctuations in academic stress and psychological distress (hypothesis 3). Fourth, the associations between academic stress, academic self-efficacy, and psychological distress will be more salient for girls than boys (hypothesis 4). Fifth, fluctuations in psychological distress will predict opposite fluctuations in subsequent academic self-efficacy (hypothesis 5). Lastly, fluctuations in academic self-efficacy will predict an opposite fluctuation in subsequent academic stress (hypothesis 6). Due to a lack of previous research on the effect of psychological distress on later academic stress, there is no specific hypothesis regarding this relationship. However, the association is investigated in the model.

## Methods

### Procedure and Participants

The participants were part of the COMPLETE project (Larsen et al., 2018), a randomised controlled trial aiming to improve the psychosocial learning environment and reduce dropout rates in upper secondary school in Norway. Sixteen schools in four municipalities agreed to participate in the study. The project randomly assigned schools to one of two intervention conditions or the control group. All students who started in August 2016 in the mentioned schools were invited to participate. The participants in this study attended a general education programme, which spans 3 years of upper secondary school from grade 11 through grade 13. The study followed a cohort of students from the beginning to the end of this education. Participants (*N* = 1508) were adolescents who had recently started in grade 11. The respondents completed surveys in August 2016 (start of grade 11), March 2017 (end of grade 11), March 2018 (end of grade 12), and March 2019 (end of grade 13). Students who were part of the same cohort, but were absent at a previous data collection, were allowed to participate in the following data collections throughout the study. Please see Table 3 for more details on the number of participants across time points.

The Norwegian Centre for Research Data (NSD) approved that the COMPLETE project is in line with GDPR. Students under age 16 needed parental/guardian consent before participating, and respondents actively consented to participate. Ahead of participation, the students were informed about the study’s aims. Researchers and research assistants in the project collected survey data using tablets on the school grounds. Students not physically present during data collection were invited to participate via e-mail.

Concerning the participant’s age at baseline, they were 15 (*n* = 425, 28.2%), 16 (*n* = 955, 63.3%), 17 (*n* = 63, 4.2%), 18 (*n* = 23, 1.5%), 19 (*n* = 15, 1%), 20 (*n* = 8, 0.5%), 21 (*n* = 11, 0.7%), 22 (*n* = 4, 0.3%), 23 (*n* = 1, 0.1%), and 24 (*n* = 3, 0.2%) years old. Regarding gender, 60.7% were girls, and 39.3% were boys. The reason for the somewhat unequal distribution of gender is that girls comprise the majority of general education students in Norway. In contrast, approximately nine out of ten students in vocational education are boys (SSB, 2022). Most students were born in Norway (70.6%), and 5.5% had an immigrant background. Concerning perceived family wealth, a median split indicated that 52.9% thought their family were in a high socioeconomic position, and 22.5% believed their family were in a low socioeconomic position.

### Instruments

#### Academic stress

The student’s academic stress was measured using a single indicator from the study ‘Health Behaviour in School-Aged Children (HBSC)’ (Klinger et al., 2015; WHO, 2012). Participants answered how stressed they felt due to the schoolwork they must do (both work during school hours and homework). The response scale ranged from 1 (not at all) to 4 (a lot).

#### Academic self-efficacy

The participants’ academic self-efficacy was assessed using the five-item academic efficacy scale from Patterns of Adaptive Learning Scales (PALS: Midgley et al., 2000). The scale is a context-specific measure of how capable individuals perceive themselves to be in performing and mastering schoolwork (i.e. classwork and homework). An item example is ‘even if the work is hard, I can learn it’. The participants responded to the items on a Likert scale ranging from 1 (not at all confident) to 5 (very confident). Earlier studies have found acceptable Cronbach’s alpha values (>0.78) (Midgley et al., 2000).

#### Psychological distress

Participants’ psychological distress was measured by the Norwegian five-item short version of the Symptom Check List-90-R, based on the anxiety and depression subscales (Tambs & Moum, 1993). This measure is not a diagnostic tool for anxiety or depression disorders but a global indicator of mixed anxiety and depressive symptoms (Siqveland et al., 2016). Adolescents assessed how bothered or distressed they had been in the last 14 days on a scale ranging from 1 (not at all) to 4 (very much). Example indicators of depression and anxiety are ‘feeling blue and sad’ and ‘feeling tense and worried’, respectively. Previous research indicates acceptable Cronbach’s values (>0.83) (Gjerde et al., 2011; Skrove et al., 2013; Strand et al., 2003; Tambs & Moum, 1993).

#### Gender

Gender was retrieved from registry data, coded as 0 (boys) and 1 (girls). Of note, participants also answered a question on gender identification (female, male, or other) in the questionnaires. However, very few respondents identified as non-cis or other-gendered (14 respondents on baseline). Thus, multigroup comparisons were not viable using all groups (cis females, cis males, non-cis females, non-cis males, and other-gendered).

#### Control variables

The following variables were included as time-invariant covariates in the model. Two dummy variables were created based on intervention conditions —participants were either in an intervention group (coded as 1) or not (coded as 0). The study measured socioeconomic position using a single indicator question on perceived family wealth (Iversen & Holsen, 2008), which was dummy coded as 0 (low) and 1 (high) by a median split. Regarding country of origin, Norwegian-born participants were coded as 0, and participants born outside of Norway were coded as 1.

### Analytical Plan

#### Preliminary analyses

Initial analyses investigated omega reliability, descriptive statistics, and correlations using SPSS version 28 (IBM corp, 2021). M*plus* version 8 (Muthén & Muthén, 1998–2017) and maximum likelihood (ML) estimation were used for structural equation modelling (SEM). Several criteria were used to assess the model fit of the SEM models. Model fit was considered acceptable if CFI > 0.90, RMSEA < 0.08, and SRMR < 0.08 (Byrne, 2012; Hu & Bentler, 1999). When investigating measurement invariance, the following fit criteria were used between comparison and nested models: ΔCFI < 0.010, ΔRMSEA < 0.015, and ΔSRMR < 0.030 (Chen, 2007).

This study investigated measurement invariance across time and gender using the effects-coding approach by Little et al. (2006), which is preferable to other methods (Breitsohl, 2019). In effects-coding, the average factor loadings across all indicators are constrained to 1.0, and the sum of the indicator intercepts is constrained to 0.0. The configural models were otherwise freely estimated. Equal factor loading constraints were applied across time and gender to establish metric (weak) invariance for the multiple indicator RI-CLPM (Hamaker, 2018). The invariance constraints were retained in further modelling. The academic self-efficacy and psychological distress scales achieved partial weak invariance, wherein at least two indicators of each scale were invariant over time and gender (Byrne et al., 1989). For space constraints, the measurement invariance results are presented in Table 2.

#### Primary analyses

The random intercept cross-lagged panel model (RI-CLPM) with academic stress, academic self-efficacy, and psychological distress was modelled following the approaches by Hamaker (2018) and Mulder and Hamaker (2021). First, each construct’s random intercept (interindividual, trait-like components) was specified by adding regression coefficients from the intercepts to corresponding latent factors at each time point, constrained to 1.0. Second, 12 second-order latent factors (state-like components) were specified (one latent factor for each of the four time points in three constructs), with regression coefficients to corresponding first-order latent factors constrained to 1.0. Third, to ensure the random intercepts and within-person variables capture all variance, the variances of the first-order latent factors were constrained to 0.0. Lastly, socioeconomic position, gender, country of origin, and intervention conditions were added as control variables in the model, regressed on the random intercepts.

**Fig. 1:**
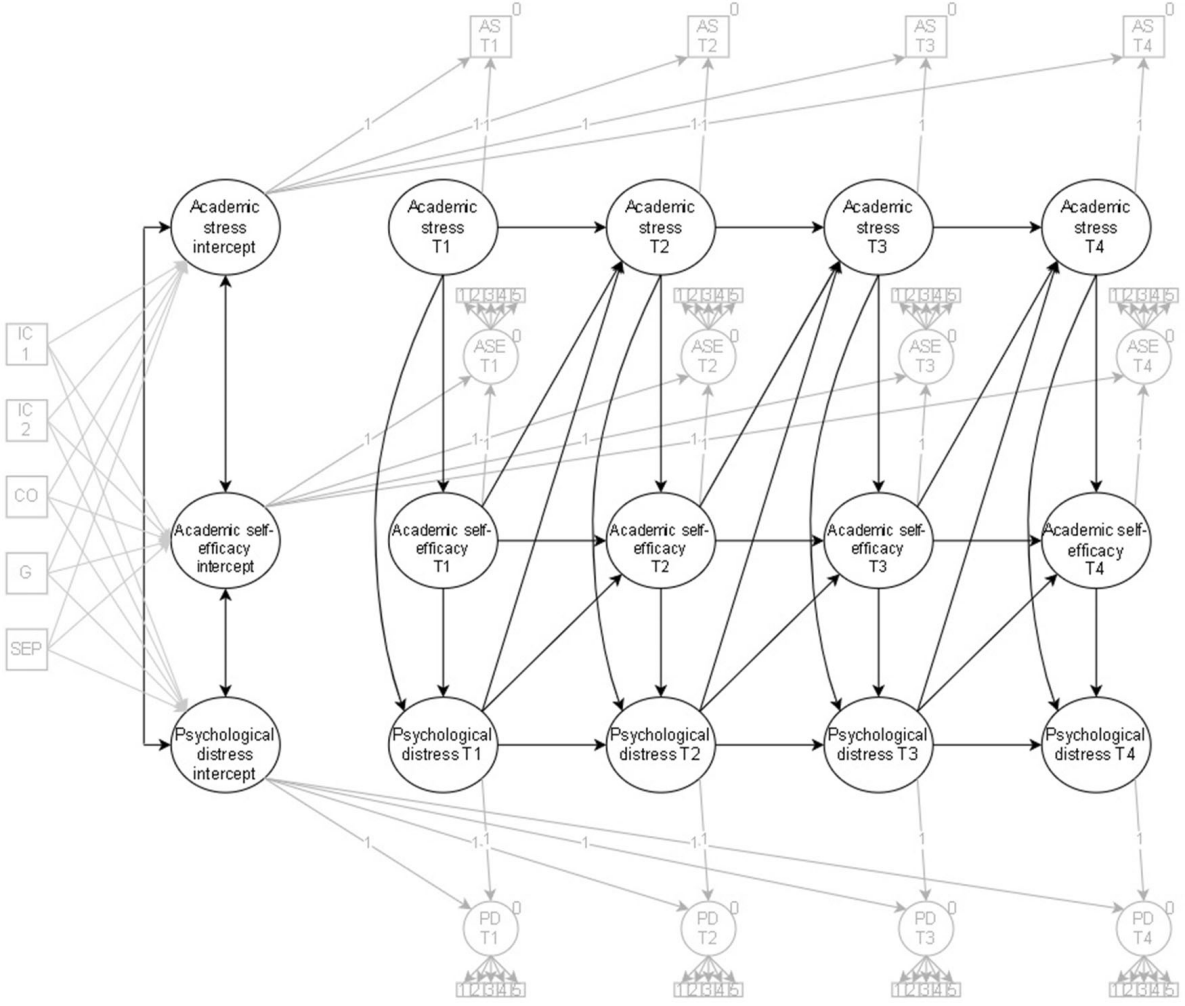
Model Specification of the Random Intercept Cross-lagged Panel Model of Academic Stress, Academic Self-efficacy, and Psychological Distress. IC = intervention condition, CO = country of origin, G = gender, SEP = socioeconomic position, PD = psychological distress, ASE = academic self-efficacy, AS = academic stress

Academic stress was specified as a predictor of concurrent academic self-efficacy and psychological distress on an intraindividual level throughout the study period (see Fig. 1 for model specification), mainly because the first and second stress appraisals happen roughly simultaneously within individuals (Lazarus & Folkman, 1984). Further, the effect of academic self-efficacy on later academic stress and the impact of psychological distress on subsequent academic self-efficacy and academic stress was examined. A freely estimated model was compared to a time-invariant model (i.e. coefficients are equal over time). The time-invariant constraints were retained if the model fit did not significantly deteriorate the chi-square. If the constraints significantly deteriorated model fit, the constraints were not tenable and removed. Next, this study examined the academic self-efficacy mediation between academic stress and psychological distress using the “model indirect” syntax in M*plus*.

To investigate if gender moderated the effects in the RI-CLPM, time-invariant constraints were initially investigated for both genders separately. Then, a multigroup analysis on the RI-CLPM with 1000 bootstraps using gender as a grouping variable was conducted, and the model constraint function in M*plus* was used to compare estimates across groups.

### Missingness

According to Little’s missing completely at random (MCAR) test, the patterns of missingness in the study’s variables were completely random (*χ*2 = 3092.302, df = 3031, *p* = 0.215). Full information maximum likelihood (FIML) was used to handle potential missing data at the construct level (Newman, 2014). Detailed information regarding the number of respondents across time is in Table 3.

## Results

### Descriptive Statistics

The descriptive statistics of the study variables are presented in Table 1. There were significant gender differences in all variables. Girls experienced significantly higher academic stress, psychological distress, and lower academic self-efficacy at all times than boys. The gender differences in terms of effect sizes were, according to Cohen (1988), moderate to large concerning academic stress, moderate regarding psychological distress, and negligible to small concerning academic self-efficacy.

**Table 1:** Descriptive statistics of the study variables

				Gender			
	*N*	*ω*	Min–Max	Girls *M* (*SD*)	Boys *M* (*SD*)	*p* value	*d*
Academic stress T1	1110	–	1–4	2.72 (0.84)	2.18 (0.82)	<0.001	−0.65
Academic stress T2	1153	–	1–4	2.95 (0.85)	2.35 (0.91)	<0.001	−0.69
Academic stress T3	930	–	1–4	3.07 (0.87)	2.45 (0.93)	<0.001	−0.70
Academic stress T4	953	–	1–4	3.37 (0.76)	2.69 (0.85)	<0.001	−0.85
Psychological distress T1	1114	0.90	1–4	2.00 (0.78)	1.55 (0.67)	<0.001	−0.63
Psychological distress T2	1147	0.90	1–4	2.17 (0.82)	1.60 (0.63)	<0.001	−0.76
Psychological distress T3	926	0.90	1–4	2.20 (0.80)	1.70 (0.71)	<0.001	−0.65
Psychological distress T4	994	0.89	1–4	2.28 (0.81)	1.88 (0.28)	<0.001	−0.51
Academic self-efficacy T1	1085	0.91	1–5	4.04 (0.72)	4.14 (0.78)	0.030	0.14
Academic self-efficacy T2	1151	0.91	1–5	3.92 (0.80)	4.12 (0.75)	<0.001	0.26
Academic self-efficacy T3	923	0.92	1–5	3.88 (0.85)	4.09 (0.79)	<0.001	0.26
Academic self-efficacy T4	947	0.89	1–5	3.65 (0.98)	3.79 (1.04)	0.037	0.14

d = Cohen’s d, *ω* = omega reliability

### Random Intercept Cross-lagged Panel Model of Academic Stress, Academic Self-efficacy, and Psychological Distress

**Fig. 2:**
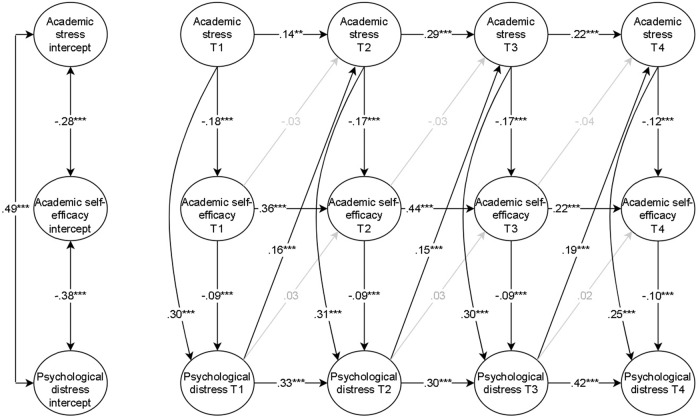
Random Intercept Cross-lagged Panel Model of Academic Stress, Academic Self-efficacy, and Psychological Distress. Standardised estimates are presented. The grey lines are non-significant. *** *p* < 0.001, ** *p* < 0.01

The RI-CLPM of academic stress, academic self-efficacy, and psychological distress produced good model fit: *χ*^2^ = 2241.786, df = 1031, *p* < 0.001, RMSEA [95% CI] = 0.032 [0.030, 0.034], CFI = 0.954, SRMR = 0.039. The model included metric invariance constraints and socioeconomic position, country of origin, gender, and intervention conditions as time-invariant covariates. Next, a fully time-invariant model with identical constraints on the regression coefficients over time was investigated. A chi-square difference test showed that the model fit significantly deteriorated (Δ*χ*^2^ = 40.658, Δdf = 21, *p* = 0.006). The autoregressive constraints were removed, and the time-invariant, cross-lagged constraint model was compared to the freely estimated model. The model fit did not significantly deteriorate: Δ*χ*^2^ = 24.326 Δdf = 15, *p* = 0.060. Therefore, the constraints were deemed tenable, and the partially time-invariant model produced good fit (*χ*^2^ = 2266.112, df = 1046, *p* < 0.001, RMSEA [95% CI] = 0.032 [0.030, 0.034], CFI = 0.954, SRMR = 0.040). The results are presented in Fig. 2, and more details are in table 4.

In support of hypothesis one, the correlation between academic stress and psychological distress was positive and moderate in effect size at the interindividual level (i.e. the random intercepts) (*r* = 0.49, *p* < 0.001). Moreover, the interindividual association between psychological distress and academic self-efficacy was negative and moderate (*r* = −0.38, *p* < 0.001). Lastly, the correlation between academic self-efficacy and academic stress intercepts was negative and small (*r* = −0.28, *p* < 0.001). Thus, adolescents who experienced high academic stress throughout their upper secondary school education were also likely to experience high psychological distress and low academic self-efficacy during the same time. Additionally, individuals likely experienced opposite levels of psychological distress and academic self-efficacy during this period.

The autoregressive regression coefficients were positive and significant in academic stress from T1 to T2 (*β* = 0.14, *p* < 0.01), T2 to T3 (*β* = 0.29, *p* < 0.001), and T3 to T4 (*β* = 0.22, *p* < 0.001). Similarly, there were positive and significant carry-over stability effects in academic self-efficacy from T1 to T2 (*β* = 0.36, *p* < 0.001), T2 to T3 (*β* = 0.44, *p* < 0.001), and T3 to T4 (*β* = 0.22, *p* < 0.001). Lastly, fluctuations in psychological distress were positively and significantly associated with later fluxes in psychological distress from T1 to T2 (*β* = 0.33, *p* < 0.001), T2 to T3 (*β* = 0.30, *p* < 0.001), and T3 to T4 (*β* = 0.42, *p* < 0.001). Thus, adolescents were increasingly likely to experience similar fluctuations at approximate time points in all three constructs.

In support of hypothesis two, individuals with a deviating level of academic stress were increasingly likely to experience the opposite deviation in concurrent academic self-efficacy on T1 (*β* = −0.18, *p* < 0.001), T2 (*β* = −0.17, *p* < 0.001), T3 (*β* = −0.17, *p* < 0.001), and T4 (*β* = −0.12, *p* < 0.001). In addition, fluctuations in academic stress were positively and significantly related to changes in concurrent psychological distress on T1 (*β* = 0.30, *p* < 0.001), T2 (*β* = 0.31, *p* < 0.001), T3 (*β* = 0.30, *p* < 0.001), and T4 (*β* = 0.25, *p* < 0.001).

Fluctuations in academic self-efficacy were predictive of oppositional fluctuations in concurrent psychological distress on T1 (*β* = −0.09, *p* < 0.001), T2 (*β* = −0.09, *p* < 0.001), T3 (*β* = −0.09, *p* < 0.001), and T4 (*β* = −0.10, *p* < 0.001). Supporting hypothesis three, the results showed that academic self-efficacy partially mediated the time-invariant association between concurrent academic stress and psychological distress on T1 (*β* = 0.02, *p* < 0.01), T2 (*β* = 0.02, *p* < 0.01), T3 (*β* = 0.02, *p* < 0.01), and T4 (*β* = 0.01, *p* < 0.01).

There was no support for hypotheses five or six. The results indicated a null effect between psychological distress and later academic self-efficacy. Similarly, academic self-efficacy did not impact later academic stress. However, the impact of psychological distress on subsequent academic stress was positive from T1 to T2 (*β* = 0.16, *p* < 0.001), T2 to T3 (*β* = 0.15, *p* < 0.001), and T3 to T4 (*β* = 0.19, *p* < 0.001). Thus, fluctuations in psychological distress were consistently associated with similar fluxes in academic stress approximately 1 year later throughout the study.

#### Gender moderation model

Before the moderation analysis of the RI-CLPM of academic stress, academic self-efficacy, and psychological distress, the appropriateness of the time-invariant constraints enforced in the mediation model was separately examined for boys and girls. The chi-square in the freely estimated RI-CLPMs was compared to the chi-square in the time-invariant constraint models in both genders. The chi-square difference tests were non-significant for both genders (*p* > 0.05), indicating that the time-invariant constraints were tenable. Thus, the following nine parameters between boys and girls were compared: three intercept correlation coefficients and six time-invariant regression coefficients (academic stress on concurrent academic self-efficacy and psychological distress; academic self-efficacy on concurrent psychological distress; psychological distress on subsequent academic self-efficacy and academic stress; academic self-efficacy on subsequent academic stress).

**Fig. 3:**
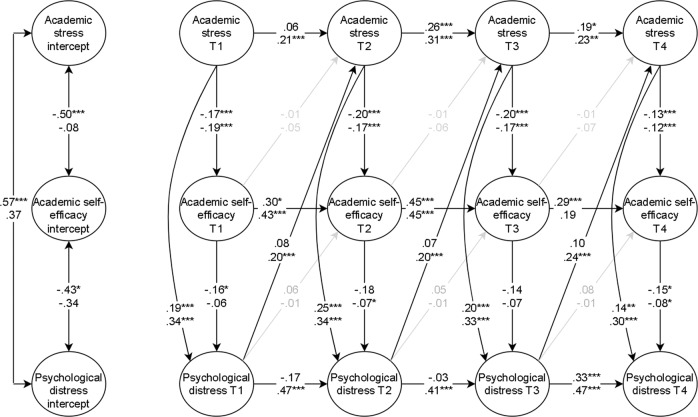
Random Intercept Cross-lagged Panel Model of Academic Stress, Academic Self-efficacy, and Psychological Distress Moderated by Gender. Boys on the upper line and girls on the lower line. Standardised estimates are presented. The grey lines are non-significant. *** *p* < 0.001, ** *p* < 0.01, * *p* < 0.05

The gender moderation RI-CLPM of academic stress, academic self-efficacy, and psychological distress achieved acceptable model fit: *χ*^2^ = 3727.383, df = 2059, *p* < 0.001, RMSEA [95% CI] = 0.038 [0.036, 0.040], CFI = 0.933, SRMR = 0.057. The results are presented in Fig. 3 and table 5. In partial support of the fourth hypothesis, three parameters significantly differed across gender: the intercept correlation between academic stress and academic self-efficacy (*r*_difference_ = 0.086, *p* = 0.025), the intercept correlation between psychological distress and academic stress (*r*_difference_ = –0.082, *p* = 0.044), and the time-invariant regression coefficient from academic stress to concurrent psychological distress (B_difference_ = 0.164, *p* = 0.000). Of note, the difference tests consider unstandardised estimates, while Fig. 3 shows the standardised results. Please see table 5 for further details on model estimates.

The significance of the indirect effects of academic stress on concurrent psychological distress through academic self-efficacy disappeared in the moderation analysis. There were no apparent gender differences in these effects (see table 6 for details). However, the 95% confidence interval of the indirect effect did not include zero for both genders. Thus, the mediation effect, albeit small, might still be relevant for both genders despite the lack of a significant *p* value.

There was a significantly stronger intercept correlations between academic stress and psychological distress for boys (*r* = 0.57, *p* < 0.001) than girls (*r* = 0.37, *p* > 0.05). Additionally, the interindividual association between academic stress and academic self-efficacy was significantly stronger for boys (*r* = −0.50, *p* < 0.001) than girls (*r* = −0.08, *p* > 0.05). Hence, boys who experienced a high (or low) level of academic stress in late secondary school were more likely to experience a similar level of psychological distress and oppositional level academic self-efficacy during the same time compared to girls. Girls had significantly larger direct effects from academic stress to concurrent psychological distress (T1: *β* = 0.34, *p* < 0.001; T2: *β* = 0.34, *p* < 0.001; T3: *β* = 0.33, *p* < 0.001; and T4: *β* = 0.30, *p* < 0.001) than boys (T1: *β* = 0.19, *p* < 0.001; T2: *β* = 0.25, *p* < 0.001; T3: *β* = 0.20, *p* < 0.001; and T4: *β* = 0.14, *p* < 0.001). Thus, girls with unusually high (or low) academic stress at each time point were more likely to experience unusually high (or low) psychological distress concurrently than boys.

### Sensitivity Analyses

This study investigated several competing models, such as different time lags between the constructs, and examined the impact of missingness on the selected model. The final model was chosen because (1) the theoretical assumptions of the transactional theory of stress and coping argue that the first and second appraisals occur simultaneously, and (2) the AIC and BIC values in the final model were lower than competing models. Regarding missingness, the final model was compared across three groups in our sample: participants with complete data (no missingness), participants with intermittent missing data patterns (non-dropouts), and all participants. The models produced similar patterns of results in terms of coefficients and standard errors.

## Discussion

Few or none have investigated the associations between academic stress and psychological distress while separating inter- and intrapersonal effects. Consequently, there is little knowledge of possible explanatory mechanisms or moderators in the mentioned association on an intraindividual level. This study sought to fill that knowledge gap. The results implied that, during upper secondary school, the normative levels of academic stress, academic self-efficacy, and psychological distress were associated. Further, that academic stress consistently predicted psychological distress throughout the study and that academic self-efficacy partially mediated this relationship. Recursively, psychological distress impacted later academic stress. Lastly, the intraindividual association between academic stress and psychological distress was stronger for girls, while the interpersonal associations between academic stress, academic self-efficacy, and psychological distress were stronger for boys.

### The Longitudinal Associations Between Academic Stress, Academic self-efficacy, and Psychological Distress

Aligning with the assumptions in the transactional theory of stress and coping (Lazarus & Folkman, 1984) and previous research, the association between academic stress and psychological distress was positive within adolescents during upper secondary school. Adolescents with, for them, unusually high (or low) academic stress at one time were increasingly likely to experience unusually high (or low) psychological distress simultaneously. Moreover, fluctuations in psychological distress were related to similar fluxes in academic stress on the following occasions. These findings indicate that interventions successful in decreasing levels of academic stress and psychological distress (e.g. Feiss et al., 2019) might lower levels in the other factor concurrently and over time, respectively. However, it might be beneficial for implementation research to investigate the effect of school-based measures on the intraindividual association between academic stress and psychological distress. For instance, are interventions designed on an interpersonal level effective in reducing unusually high academic stress or psychological distress at the intraindividual level? Such research might further important knowledge in the field.

Academic self-efficacy functioned as a mechanism, partially explaining the concurrent relationship between academic stress and psychological distress within adolescents over time. Indeed, fluctuations in academic stress were related to oppositional fluctuations in academic self-efficacy and similar fluxes in psychological distress simultaneously. This effect aligns with central assumptions on how self-efficacy changes within individuals, wherein adverse feelings in certain situations decrease self-efficacy in the same settings (Bandura, 1997). Because stress, as measured in this study, is an inherently negative affective state, the reduction in self-efficacy for the same context that induced the negative feeling has been explored in many instances (for an overview, see Usher & Pajares, 2008). However, the finding that fluctuations in academic self-efficacy partly explain changes in psychological distress during fluxes in academic stress is novel. Theoretical or conceptual models of stress and mental health problems might include this mechanism in adolescent samples. Even though the transactional theory of stress and coping (Lazarus & Folkman, 1984) and self-efficacy theory (Bandura, 1997) describe processes occurring within individuals, such as cognitive evaluations and change, and emotional responses, the frameworks have used research on the interpersonal level to postulate intraindividual psychological developments.

The impact of psychological distress on later academic stress was positive. Hence, fluctuations in psychological distress were associated with similar changes in academic stress ~1 year later throughout the study period. Little research has focused on the impact psychological distress has on academic stress, mainly because academic stress is assumed to be an antecedent in the relationship between the two (e.g. Murberg & Bru, 2005; Tian et al., 2019). However, psychologically distressed individuals often behave in manners that create situations they perceive as stressful (Hammen, 2020). The findings in this study suggest that this effect might also apply to the educational setting, particularly the perception of school- and homework as stressful. In other words, due to an unexpected rise in psychological distress, students might behave in ways that increase the likelihood of experiencing the school- and homework as stressful later. It is possible that unusually psychologically distressed students postpone or avoid the academic workload or even physically withdraw from school. Such behaviour might result in perceiving school- and homework as a *threat* instead of challenging, positive, or irrelevant to personal well-being. Thus, an adverse loop of school-related stress and hopelessness, sadness, and worry might arise.

### Gender Differences

Regarding gender differences, fluctuations in academic stress were more strongly associated with concurrent fluxes in psychological distress for girls than boys. The stronger intraindividual association for girls might be related to the academic pressure and demands girls perceive by others and themselves. For example, girls experience more pressures and expectations concerning their school performances (Gådin & Hammarström, 2000) and are more worried and affected by the beliefs and judgments of other people (Rudolph, 2002) than boys. Indeed, one report indicated that 39% of Norwegian girls, compared to 14% of boys, who experienced school-related stress “very often” also felt “very bothered” by symptoms of anxiety and depression (Eriksen et al., 2017). On the other hand, academic stress was significantly more strongly related to academic self-efficacy and psychological distress on an interindividual level throughout upper secondary school for boys than girls.

### Limitations

One limitation is that the sample is not nationally representative. However, the participants have typical characteristics of Norwegian and Western cultures, and the results are likely transferable to other late secondary school samples similar in age and demographics.

Another possible limitation is the single-item measurement of academic stress. Single-item measures have uncertain reliability and might not adequately capture a complex psychological construct (Allen et al., 2022). A latent factor with several indicators might have provided more information concerning academic stress as a construct. However, the single indicator has been validated previously and functions well as a measure of academic stress (Klinger et al., 2015). Additionally, based on comparisons with negative stress items in stress scales, such as the perceived stress scale (Cohen et al., 1983) and the educational stress scale for adolescents (Sun et al., 2011), the included indicator is expected to have strong face validity. The bivariate correlations between the indicator across time points were moderate to strong in effect size, according to Cohen (1988), ranging from *r* = 0.36 (*p* < 0.001) to *r* = 0.55 (*p* < 0.001).

Any bias associated with self-report measures, such as common method bias (Doty & Glick, 1998) or under- and overreporting (Hunt et al., 2003; Sigmon et al., 2005), might be considered another limitation, as all data was self-reported in this study. Regarding underreporting, one study found that the difference between self-reported and administrative health service data on mood and anxiety disorders has decreased over time, particularly in adolescence (O’Donnell et al., 2016). This finding might indicate improved mental health literacy or a positive societal change in the perceptions of mental health, such as reduced stigma (O’Donnell et al., 2016). Concerning common method bias, a post hoc Harman’s single factor test was performed on each time point to investigate if a latent factor was accountable for the variance in the study’s data (Chang et al., 2010). The results showed that a single factor did not account for the majority of the variance, and several factor solutions were more appropriate for each measurement occasion.

Lastly, the mediating effect of academic self-efficacy between academic stress and psychological distress was small. Therefore, caution in interpreting this finding is advised. However, within-person effects tend to be smaller than effects that include both between- and within-person variances. Furthermore, the model controls for prior levels of the predictive variables. Thus, the mediation effect is relevant even though it is small.

### Future Directions

Academic self-efficacy was only a partial mediator in the concurrent association between academic stress and psychological distress, implying it only explains parts of the relationship. Future research should include other relevant mediators between stress and psychological distress (e.g. coping mechanisms) in a school setting to further unravel these associations over time. Notably, researchers are encouraged to separate between- and within-person effects to truly parse the associations between academic stress and adolescent psychological distress. Moreover, when investigating the associations between academic stress, academic self-efficacy (or other mediators), and psychological distress, researchers should consider the effect of gender.

## Conclusion

There is a research gap on explanatory mechanisms and moderators in the intraindividual relationship between academic stress and psychological distress during adolescence. This study aimed to fill this gap. Specifically, the inter- and intraindividual associations between academic stress, academic self-efficacy, and psychological distress, and possible gender differences in these relationships, were investigated in an upper secondary school cohort. The results showed that academic stress, directly and indirectly through academic self-efficacy, impacted concurrent psychological distress consistently during 3 years in mid-late adolescence. Psychological distress systematically affected later academic stress. Intraindividual effects were more salient for girls, and interindividual effects were stronger for boys. The study findings imply the existence of an exacerbating feedback loop between academic stress and psychological distress in upper secondary school, which functions differently for boys and girls and is partly explained by fluctuations in academic self-efficacy.

## Appendix

Tables 2–6

**Table 2:** Measurement Invariance

	*χ*^2^	df	RMSEA [90% CI]	CFI	SRMR	ΔRMSEA	ΔCFI	ΔSRMR
Across time								
*Psychological distress*								
Configural	428.732	134	0.038 [0.034, 0.042]	0.978	0.027			
Metric	466.374	149	0.038 [0.034, 0.042]	0.976	0.034	0.000	0.002	0.007
*Academic self-efficacy*								
Configural	962.980	134	0.065 [0.061, 0.069]	0.945	0.035			
Metric^a^	998.133	146	0.063 [0.059, 0.067]	0.943	0.065	0.002	0.002	0.030
Across gender								
*Psychological distress*								
Configural	615.975	268	0.042 [0.037, 0.046]	0.971	0.039			
Metric^b^	663.697	287	0.042 [0.038, 0.046]	0.969	0.066	0.000	0.002	0.027
*Academic self-efficacy*								
Configural	1196.946	268	0.068 [0.065, 0.072]	0.938	0.045			
Metric^a^	1234.547	285	0.067 [0.063, 0.071]	0.937	0.063	0.001	0.001	0.018

^a^Three indicator factor loading constraints were removed for model fit

^b^Two indicator factor loading constraints were removed for model fit

**Table 3:** Respondents across measurement waves

Time point	*N*	Percent	Cumulative percent
T1	55	3.6	3.6
T2	34	2.3	5.9
T3	23	1.5	7.4
T4	138	9.2	16.6
T1 + T2	144	9.5	26.1
T1 + T3	11	0.7	26.9
T1 + T4	16	1.1	27.9
T2 + T3	35	2.3	30.2
T2 + T4	17	1.1	31.4
T3 + T4	43	2.9	34.2
T1 + T2 + T3	190	12.6	46.8
T1 + T2 + T4	155	10.3	57.1
T1 + T3 + T4	38	2.5	59.6
T2 + T3 + T4	67	4.4	64.1
T1 + T2 + T3 + T4	542	35.9	100
Total	1508	100

**Table 4:** Estimates from the random intercept cross-lagged panel model of academic stress, academic self-efficacy, and psychological distress

			Unstandardised			Standardised		
			Est.	SE	95% CI	Est.	SE	95% CI
*Autoregressive regression coefficients*								
T1 ASE	→	T2 ASE	0.382***	0.072	0.241, 0.523	0.362***	0.066	0.233, 0.491
T2 ASE	→	T3 ASE	0.464***	0.067	0.332, 0.596	0.438***	0.060	0.319, 0.556
T3 ASE	→	T4 ASE	0.264***	0.078	0.111, 0.417	0.221***	0.066	0.092, 0.350
T1 AS	→	T2 AS	0.142**	0.051	0.041, 0.243	0.136**	0.048	0.041, 0.231
T2 AS	→	T3 AS	0.306***	0.050	0.208, 0.403	0.292***	0.046	0.202, 0.381
T3 AS	→	T4 AS	0.185***	0.048	0.091, 0.280	0.218***	0.055	0.110, 0.326
T1 PD	→	T2 PD	0.325***	0.080	0.168, 0.482	0.326***	0.074	0.181, 0.471
T2 PD	→	T3 PD	0.322***	0.080	0.166, 0.479	0.300***	0.074	0.155, 0.445
T3 PD	→	T4 PD	0.439***	0.066	0.310, 0.569	0.424***	0.053	0.321, 0.527
*Time-invariant regression coefficients*								
T1 AS	→	T1 ASE	−0.136***	0.022	−0.178, −0.093	−0.176***	0.028	−0.231, −0.121
T2 AS	→	T2 ASE	−0.136***	0.022	−0.178, −0.093	−0.174***	0.028	−0.228, −0.119
T3 AS	→	T3 ASE	−0.136***	0.022	−0.178, −0.093	−0.172***	0.027	−0.225, −0.118
T4 AS	→	T4 ASE	−0.136***	0.022	−0.178, −0.093	−0.122***	0.020	−0.162, −0.082
T1 AS	→	T1 PD	0.212***	0.020	0.174, 0.251	0.296***	0.028	0.241, 0.352
T2 AS	→	T2 PD	0.212***	0.020	0.174, 0.251	0.310***	0.029	0.253, 0.368
T3 AS	→	T3 PD	0.212***	0.020	0.174, 0.251	0.302***	0.028	0.248, 0.356
T4 AS	→	T4 PD	0.212***	0.020	0.174, 0.251	0.248***	0.027	0.196, 0.300
T1 ASE	→	T1 PD	−0.079***	0.024	−0.126, −0.032	−0.085***	0.026	−0.137, −0.033
T2 ASE	→	T2 PD	−0.079***	0.024	−0.126, −0.032	−0.090***	0.028	−0.145, −0.035
T3 ASE	→	T3 PD	−0.079***	0.024	−0.126, −0.032	−0.089***	0.028	−0.143, −0.035
T4 ASE	→	T4 PD	−0.079***	0.024	−0.126, −0.032	−0.103***	0.030	−0.162, −0.043
T1 PD	→	T2 ASE	0.031	0.042	−0.052, 0.114	0.027	0.037	−0.045, 0.100
T2 PD	→	T3 ASE	0.031	0.042	−0.052, 0.114	0.026	0.035	−0.042, 0.094
T3 PD	→	T4 ASE	0.031	0.042	−0.052, 0.114	0.023	0.031	−0.038, 0.084
T1 PD	→	T2 AS	0.227***	0.054	0.121, 0.332	0.155***	0.038	0.082, 0.229
T2 PD	→	T3 AS	0.227***	0.054	0.121, 0.332	0.148***	0.038	0.073, 0.223
T3 PD	→	T4 AS	0.227***	0.054	0.121, 0.332	0.187***	0.048	0.094, 0.281
T1 ASE	→	T2 AS	−0.040	0.044	−0.127, 0.047	−0.030	0.033	−0.094, 0.035
T2 ASE	→	T3 AS	−0.040	0.044	−0.127, 0.047	−0.030	0.033	−0.095, 0.036
T3 ASE	→	T4 AS	−0.040	0.044	−0.127, 0.047	−0.037	0.042	−0.119, 0.045
*Correlation coefficients*								
PD RI	↔	ASE RI	−0.073***	0.016	−0.104, −0.043	−0.378***	0.071	−0.518, −0.238
PD RI	↔	AS RI	0.094***	0.021	0.053, 0.135	0.493***	0.066	0.363, 0.623
ASE RI	↔	AS RI	−0.052**	0.017	−0.085, −0.018	−0.280***	0.082	−0.441, −0.119
*Covariates*								
IC 1	→	PD RI	0.004	0.051	−0.095, 0.103	0.004	0.048	−0.091, 0.098
IC 1	→	ASE RI	0.049	0.051	−0.052, 0.149	0.053	0.056	−0.057, 0.162
IC 1	→	AS RI	−0.026	0.053	−0.130, 0.078	−0.025	0.050	−0.123, 0.074
IC 2	→	PD RI	0.038	0.051	−0.062, 0.139	0.036	0.048	−0.059, 0.130
IC 2	→	ASE RI	0.013	0.052	−0.089, 0.115	0.014	0.056	−0.095, 0.123
IC 2	→	AS RI	−0.020	0.054	−0.125, 0.086	−0.018	0.050	−0.117, 0.080
CO	→	PD RI	0.032	0.073	−0.111, 0.174	0.015	0.036	−0.054, 0.085
CO	→	ASE RI	−0.047	0.075	−0.195, 0.100	−0.026	0.042	−0.108, 0.056
CO	→	AS RI	−0.123	0.077	−0.275, 0.029	−0.060	0.038	−0.133, 0.014
SEP	→	PD RI	−0.215***	0.040	−0.294, −0.136	−0.190***	0.037	−0.261, −0.118
SEP	→	ASE RI	0.260***	0.041	0.179, 0.341	0.260***	0.043	0.177, 0.344
SEP	→	AS RI	−0.091*	0.042	−0.175, −0.008	−0.080*	0.039	−0.153, −0.007
G	→	PD RI	0.480***	0.038	0.405, 0.554	0.449***	0.039	0.372, 0.526
G	→	ASE RI	−0.141***	0.039	−0.240, −0.065	−0.150***	0.041	−0.231, −0.069
G	→	AS RI	0.606***	0.040	0.528, 0.684	0.565***	0.039	0.489, 0.642

*Est.* estimate, *SE* standard error, *CI* confidence interval, *RI* random intercept, *PD* psychological distress, *AS* academic stress, *ASE* academic self-efficacy, *IC* intervention condition, *CO* country of origin, *SEP* socioeconomic position, *G* gender

****p* < 0.001; ***p* < 0.01; **p* < 0.05

**Table 5:** Estimates from the random intercept cross-lagged panel model of academic stress, academic self-efficacy, and psychological distress moderated by gender

			Unstandardised			Standardised		
			Est.	SE	95% CI	Est.	SE	95% CI
BOYS								
*Autoregressive regression coefficients*								
T1 ASE	→	T2 ASE	0.273*	0.139	0.000, 0.547	0.298*	0.145	0.000, 0.570
T2 ASE	→	T3 ASE	0.480***	0.150	0.165, 0.750	0.452***	0.134	0.170, 0.686
T3 ASE	→	T4 ASE	0.371*	0.152	0.031, 0.646	0.290*	0.123	0.022, 0.523
T1 AS	→	T2 AS	0.068	0.094	−0.124, 0.256	0.063	0.085	−0.116, 0.228
T2 AS	→	T3 AS	0.272***	0.084	0.098, 0.423	0.262***	0.079	0.097, 0.400
T3 AS	→	T4 AS	0.164	0.084	−0.010, 0.316	0.193*	0.097	−0.012, 0.364
T1 PD	→	T2 PD	−0.137	0.228	−0.618, 0.321	−0.167	0.277	−0.757, 0.320
T2 PD	→	T3 PD	−0.046	0.302	−0.800, 0.416	−0.034	0.211	−0.507, 0.341
T3 PD	→	T4 PD	0.391**	0.131	0.143, 0.645	0.332***	0.102	0.122, 0.508
*Time-invariant regression coefficients*								
T1 AS	→	T1 ASE	−0.150***	0.042	−0.233, −0.069	−0.167***	0.047	−0.256, −0.078
T2 AS	→	T2 ASE	−0.150***	0.042	−0.233, −0.069	−0.200***	0.057	−0.317, −0.093
T3 AS	→	T3 ASE	−0.150***	0.042	−0.233, −0.069	−0.196***	0.053	−0.296, −0.089
T4 AS	→	T4 ASE	−0.150***	0.042	−0.233, −0.069	−0.130***	0.037	−0.204, −0.056
T1 AS	→	T1 PD	0.111***	0.034	0.046, 0.178	0.191***	0.057	0.075, 0.299
T2 AS	→	T2 PD	0.111***	0.034	0.046, 0.178	0.254***	0.070	0.118, 0.394
T3 AS	→	T3 PD	0.111***	0.034	0.046, 0.178	0.196***	0.058	0.081, 0.308
T4 AS	→	T4 PD	0.111***	0.034	0.046, 0.178	0.142**	0.048	0.052, 0.243
T1 ASE	→	T1 PD	−0.104*	0.050	−0.213, −0.013	−0.160*	0.081	−0.333, −0.018
T2 ASE	→	T2 PD	−0.104*	0.050	−0.213, −0.013	−0.179	0.106	−0.442, −0.020
T3 ASE	→	T3 PD	−0.104*	0.050	−0.213, −0.013	−0.141	0.073	−0.293, −0.017
T4 ASE	→	T4 PD	−0.104*	0.050	−0.213, −0.013	−0.153*	0.071	−0.296, −0.021
T1 PD	→	T2 ASE	0.086	0.111	−0.138, 0.314	0.061	0.077	−0.096, 0.206
T2 PD	→	T3 ASE	0.086	0.111	−0.138, 0.314	0.047	0.066	−0.069, 0.186
T3 PD	→	T4 ASE	0.086	0.111	−0.138, 0.314	0.050	0.064	−0.074, 0.179
T1 PD	→	T2 AS	0.156	0.118	−0.075, 0.394	0.083	0.063	−0.046, 0.211
T2 PD	→	T3 AS	0.156	0.118	−0.075, 0.394	0.066	0.054	−0.024, 0.186
T3 PD	→	T4 AS	0.156	0.118	−0.075, 0.394	0.104	0.080	−0.051, 0.266
T1 ASE	→	T2 AS	−0.011	0.080	−0.169, 0.144	−0.009	0.066	−0.143, 0.108
T2 ASE	→	T3 AS	−0.011	0.080	−0.169, 0.144	−0.008	0.059	−0.126, 0.098
T3 ASE	→	T4 AS	−0.011	0.080	−0.169, 0.144	−0.010	0.073	−0.168, 0.119
*Correlation coefficients*								
PD RI	↔	ASE RI	−0.080**	0.025	−0.122, −0.032	−0.428*	0.175	−0.923, −0.222
PD RI	↔	AS RI	0.132***	0.025	0.078, 0.180	0.572***	0.085	0.420, 0.764
ASE RI	↔	AS RI	−0.100**	0.031	−0.155, −0.029	−0.498**	0.193	−0.959, −0.244
*Covariates*								
IC 1	→	PD RI	−0.085	0.070	−0.222, 0.051	−0.085	0.069	−0.221, 0.051
IC 1	→	ASE RI	0.091	0.080	−0.065, 0.247	0.104	0.092	−0.075, 0.284
IC 1	→	AS RI	−0.270***	0.077	−0.421, −0.119	−0.250***	0.072	−0.392, −0.108
IC 2	→	PD RI	0.134*	0.069	−0.312, 0.002	−0.134	0.069	−0.269, 0.001
IC 2	→	ASE RI	0.121	0.079	−0.035, 0.276	0.138	0.091	−0.040, 0.316
IC 2	→	AS RI	−0.291***	0.076	−0.441, −0.142	−0.271***	0.072	−0.411, −0.131
CO	→	PD RI	0.100	0.107	−0.110, 0.309	0.050	0.054	−0.055, 0.155
CO	→	ASE RI	−0.106	0.121	−0.343, 0.131	−0.061	0.070	−0.198, 0.076
CO	→	AS RI	−0.245	0.133	−0.506, 0.015	−0.114	0.062	−0.236, 0.007
SEP	→	PD RI	−0.334***	0.060	−0.451, −0.216	−0.291***	0.050	−0.390, −0.193
SEP	→	ASE RI	0.312***	0.069	0.177, 0.448	0.312**	0.076	0.164, 0.461
SEP	→	AS RI	−0.275***	0.066	−0.405, −0.145	−0.223***	0.055	−0.332, −0.115
GIRLS								
*Autoregressive regression coefficients*								
T1 ASE	→	T2 ASE	0.510***	0.118	0.231, 0.689	0.431***	0.108	0.182, 0.603
T2 ASE	→	T3 ASE	0.466***	0.135	0.183, 0.699	0.445***	0.115	0.184, 0.636
T3 ASE	→	T4 ASE	0.215	0.114	−0.027, 0.415	0.190	0.102	−0.025, 0.379
T1 AS	→	T2 AS	0.210***	0.066	0.069, 0.331	0.205***	0.063	0.068, 0.322
T2 AS	→	T3 AS	0.324***	0.071	0.182, 0.456	0.311***	0.066	0.179, 0.435
T3 AS	→	T4 AS	0.195**	0.074	0.035, 0.333	0.230**	0.084	0.043, 0.382
T1 PD	→	T2 PD	0.487***	0.082	0.297, 0.629	0.472***	0.076	0.304, 0.602
T2 PD	→	T3 PD	0.431***	0.093	0.236, 0.610	0.410***	0.086	0.225, 0.562
T3 PD	→	T4 PD	0.447***	0.088	0.264, 0.615	0.473***	0.068	0.324, 0.589
*Time-invariant regression coefficients*								
T1 AS	→	T1 ASE	−0.133***	0.028	−0.185, −0.076	−0.192***	0.040	−0.274, −0.116
T2 AS	→	T2 ASE	−0.133***	0.028	−0.185, −0.076	−0.166***	0.034	−0.233, −0.097
T3 AS	→	T3 ASE	−0.133***	0.028	−0.185, −0.076	−0.165***	0.035	−0.234, −0.098
T4 AS	→	T4 ASE	−0.133***	0.028	−0.185, −0.076	−0.124***	0.027	−0.176, −0.071
T1 AS	→	T1 PD	0.275***	0.026	0.225, 0.323	0.341***	0.037	0.268, 0.409
T2 AS	→	T2 PD	0.275***	0.026	0.225, 0.323	0.337***	0.038	0.254, 0.413
T3 AS	→	T3 PD	0.275***	0.026	0.225, 0.323	0.334***	0.037	0.261, 0.408
T4 AS	→	T4 PD	0.275***	0.026	0.225, 0.323	0.299***	0.040	0.230, 0.391
T1 ASE	→	T1 PD	−0.072*	0.035	−0.140, −0.007	−0.062	0.032	−0.135, −0.006
T2 ASE	→	T2 PD	−0.072*	0.035	−0.140, −0.007	−0.071*	0.035	−0.141, −0.006
T3 ASE	→	T3 PD	−0.072*	0.035	−0.140, −0.007	−0.070	0.036	−0.143, −0.006
T4 ASE	→	T4 PD	−0.072*	0.035	−0.140, −0.007	−0.084*	0.039	−0.161, −0.009
T1 PD	→	T2 ASE	−0.014	0.047	−0.107, 0.078	−0.013	0.047	−0.111, 0.076
T2 PD	→	T3 ASE	−0.014	0.047	−0.107, 0.078	−0.013	0.046	−0.110, 0.076
T3 PD	→	T4 ASE	−0.014	0.047	−0.107, 0.078	−0.012	0.042	−0.101, 0.068
T1 PD	→	T2 AS	0.250***	0.062	0.121, 0.361	0.198***	0.048	0.097, 0.286
T2 PD	→	T3 AS	0.250***	0.062	0.121, 0.361	0.196***	0.050	0.095, 0.288
T3 PD	→	T4 AS	0.250***	0.062	0.121, 0.361	0.243***	0.063	0.119, 0.362
T1 ASE	→	T2 AS	−0.074	0.057	−0.179, 0.045	−0.050	0.040	−0.127, 0.032
T2 ASE	→	T3 AS	−0.074	0.057	−0.179, 0.045	−0.057	0.044	−0.144, 0.035
T3 ASE	→	T4 AS	−0.074	0.057	−0.179, 0.045	−0.070	0.056	−0.181, 0.042
*Correlation coefficients*								
PD RI	↔	ASE RI	−0.054*	0.026	−0.105, −0.002	−0.335	0.267	−0.829, −0.024
PD RI	↔	AS RI	0.050	0.033	−0.015, 0.112	0.369	0.258	−0.198, 0.765
ASE RI	↔	AS RI	−0.013	0.025	−0.060, 0.033	−0.082	0.184	−0.376, 0.296
*Covariates*								
IC 1	→	PD RI	0.082	0.067	−0.050, 0.213	0.108	0.091	−0.070, 0.286
IC 1	→	ASE RI	0.029	0.067	−0.102, 0.159	0.032	0.073	−0.111, 0.175
IC 1	→	AS RI	0.127*	0.062	0.005, 0.248	0.168	0.083	0.005, 0.330
IC 2	→	PD RI	0.154*	0.069	0.020, 0.289	0.199	0.095	0.012, 0.385
IC 2	→	ASE RI	−0.041	0.068	−0.176, 0.093	−0.044	0.073	−0.188, 0.100
IC 2	→	AS RI	0.141*	0.064	0.016, 0.266	0.182	0.083	0.019, 0.345
CO	→	PD RI	0.005	0.095	−0.182, 0.191	0.003	0.065	−0.125, 0.131
CO	→	ASE RI	0.002	0.096	−0.186, 0.190	0.001	0.055	−0.106, 0.108
CO	→	AS RI	−0.060	0.096	−0.248, 0.129	−0.041	0.066	−0.171, 0.089
SEP	→	PD RI	−0.147**	0.051	−0.182, 0.191	−0.184	0.072	−0.325, −0.042
SEP	→	ASE RI	0.237***	0.051	0.137, 0.337	0.246*	0.054	0.140, 0.353
SEP	→	AS RI	−0.024	0.050	−0.122, 0.073	−0.030	0.063	−0.153, 0.092

*Est.* estimate, *SE* standard error, *CI* confidence interval, *RI* random intercept, *PD* psychological distress, *AS* academic stress, *ASE* academic self-efficacy, *IC* intervention condition, *CO* country of origin, *SEP* socioeconomic position, *G* gender

****p* < 0.001; ***p* < 0.01; **p* < 0.05

**Table 6:** Moderated mediation model of academic stress, academic self-efficacy, and psychological distress at the within-person level

	Academic stress → Academic self-efficacy → Psychological distress					
	*β*	SE	95% CI	B	SE	95% CI
*Total sample*						
T1	0.015**	0.005	0.005, 0.025	0.011**	0.004	0.003, 0.018
T2	0.016**	0.006	0.005, 0.027	0.011**	0.004	0.003, 0.018
T3	0.015**	0.005	0.005, 0.026	0.011**	0.004	0.003, 0.018
T4	0.013**	0.004	0.004, 0.021	0.011**	0.004	0.003, 0.018
*Boys*						
T1	0.027	0.014	0.002, 0.060	0.016	0.009	0.001, 0.036
T2	0.036	0.023	0.002, 0.092	0.016	0.009	0.001, 0.036
T3	0.028	0.016	0.002, 0.066	0.016	0.009	0.001, 0.036
T4	0.020	0.011	0.002, 0.047	0.016	0.009	0.001, 0.036
*Girls*						
T1	0.012	0.006	0.001, 0.027	0.010	0.005	0.001, 0.020
T2	0.012	0.006	0.002, 0.026	0.010	0.005	0.001, 0.020
T3	0.012	0.006	0.001, 0.025	0.010	0.005	0.001, 0.020
T4	0.010	0.005	0.001, 0.022	0.010	0.005	0.001, 0.020

*β* standardised estimate, *B* unstandardised estimate, *SE* standard error, *CI* confidence interval

***p* < 0.01
